# An Electrophysiological and Pharmacological Study of the Properties of Human iPSC-Derived Neurons for Drug Discovery

**DOI:** 10.3390/cells10081953

**Published:** 2021-07-31

**Authors:** Robert F. Halliwell, Hamed Salmanzadeh, Leanne Coyne, William S. Cao

**Affiliations:** Department of Physiology & Pharmacology, Thomas J. Long School of Pharmacy, University of the Pacific, Stockton, CA 95211, USA; hsalmanzadehdozdabi@pacific.edu (H.S.); lcoyne@chsu.edu (L.C.); wscao@pacific.edu (W.S.C.)

**Keywords:** neurotransmitter receptors, ion channels, multi-electrode array, patch-clamp

## Abstract

Human stem cell-derived neurons are increasingly considered powerful models in drug discovery and disease modeling, despite limited characterization of their molecular properties. Here, we have conducted a detailed study of the properties of a commercial human induced Pluripotent Stem Cell (iPSC)-derived neuron line, iCell [GABA] neurons, maintained for up to 3 months in vitro. We confirmed that iCell neurons display neurite outgrowth within 24 h of plating and label for the pan-neuronal marker, βIII tubulin within the first week. Our multi-electrode array (MEA) recordings clearly showed neurons generated spontaneous, spike-like activity within 2 days of plating, which peaked at one week, and rapidly decreased over the second week to remain at low levels up to one month. Extracellularly recorded spikes were reversibly inhibited by tetrodotoxin. Patch-clamp experiments showed that iCell neurons generated spontaneous action potentials and expressed voltage-gated Na and K channels with membrane capacitances, resistances and membrane potentials that are consistent with native neurons. Our single neuron recordings revealed that reduced spiking observed in the MEA after the first week results from development of a dominant inhibitory tone from GABAergic neuron circuit maturation. GABA evoked concentration-dependent currents that were inhibited by the convulsants, bicuculline and picrotoxin, and potentiated by the positive allosteric modulators, diazepam, chlordiazepoxide, phenobarbital, allopregnanolone and mefenamic acid, consistent with native neuronal GABA_A_ receptors. We also show that glycine evoked robust concentration-dependent currents that were inhibited by the neurotoxin, strychnine. Glutamate, AMPA, Kainate and NMDA each evoked concentration-dependent currents in iCell neurons that were blocked by their selective antagonists, consistent with the expression of ionotropic glutamate receptors. The NMDA currents required the presence of the co-agonist glycine and were blocked in a highly voltage-dependent manner by Mg^2+^ consistent with the properties of native neuronal NMDA receptors. Together, our data suggest that such human iPSC-derived neurons may have significant value in drug discovery and development and may eventually largely replace the need for animal tissues in human biomedical research.

## 1. Introduction

Galvani (1737–1798) and Volta (1745–1827) in the late eighteenth century established bioelectricity and the field of electrophysiology [1]. The instruments used to investigate animal electricity have since evolved radically from the differential rheotome built by Bernstein (1868) to the patch-clamp technique invented by Nobel Laureates, Neher and Sakmann (1976). Notwithstanding these major advances in technology, studies of the nervous system have relied almost exclusively on animal-derived tissues (particularly the frog excised nerve-muscle preparation) even when the primary questions have revolved around human brain functions. Indeed, when mice and rats are included, over 100 million animals are used annually in biomedical research worldwide [2].

Electrophysiological and neuropharmacological studies of human neurons have, until recently, been technically, legally and ethically difficult and often conducted on small sections (slices) of neocortex resected from patients undergoing neurosurgery to treat epilepsy, or to remove arterio-vascular malformations or neoplasms (e.g., [3]). This model retains some of the complexity of neural networks and cell diversity but may or may not represent the ‘normal’ properties of human neurons. Alternative approaches have used recombinant human receptors and ion channels expressed in human cell lines or Xenopus oocytes to investigate receptor pharmacology or drug interactions on these systems (e.g., [4]) but the complex neural networks and diverse neuronal cell types are absent in this approach.

Significant advances in stem cell technology over the last decade are now providing neural cells from diverse human stem cell populations including embryonic, fetal, adult, induced pluripotent stem cells (hiPSCs) and from the direct conversion of terminally differentiated fibroblasts into induced neurons, termed iNeurons (for review see [5]). Human stem cell derived neurons are therefore increasingly advanced as powerful new tools in drug discovery [6] despite their characterization often being limited to changes in cell morphology, immunocytochemical labeling and/or a description of minimal electrophysiological properties [5]. There are now several commercially available human stem cell lines and iPSC-derived neural cells, including iCell neurons (renamed iCell GABA Neurons, Fujifilm Cellular Dynamics Inc. FCDI). iCells neurons are a pure population of human neurons derived from IPSCs using proprietary differentiation and purification protocols and terminally differentiated into mature, cortical forebrain-like neurons [7,8]. Based on flow cytometry, gene expression and immunolabeling they are 85–90% GABAergic neurons and 10–15% glutamatergic neurons; there are no glial cells present (FCDI). In an early report, these human iCell neurons were shown to express voltage-gated sodium, potassium and L-type calcium channels and GABA evoked responses that were inhibited by bicuculline [9,10,11]. Using fluorescence-based calcium imaging, Dage and colleagues (2014) also reported the expression of AMPA and NMDA receptors in iCell neurons [7]. In the present study, we have conducted a more complete characterization of the neuropharmacological properties of iCell neurons to determine their value in drug receptor studies. Specifically, we have utilized multi-electrode arrays (MEA) and conventional patch-clamp electrophysiology, along with microscopy and immunocytochemistry to determine the expression and properties of voltage- and ligand-gated ion channels, as well as the development of synaptic activity in iCell neurons maintained for up to 3 months in vitro.

## 2. Materials and Methods

### 2.1. Cell Culture

Frozen vials of iCell Neurons (Fujifilm Cellular Dynamics Inc. [FCDI], Madison, WI, USA) were thawed as per the manufacturer’s instructions and, in preparation for immunocytochemistry and patch-clamp electrophysiology, seeded at a density of 75,000 cells per well onto 12 mm glass coverslips in 24-well plates. Prior to cell seeding, coverslips were coated with 0.01% poly-l-ornithine (Sigma-Aldrich, St. Louis, MO, USA) for 1 h at room temperature, washed twice with phosphate-buffered saline (PBS) and coated with a 3.3 μg/mL solution of laminin (Sigma-Aldrich) for 1 h in a 37 °C incubator. The laminin was aspirated immediately before the addition of cell suspensions. Cells were maintained in a 37 °C incubator (5% CO_2_/95% humidified air) in iCell Neurons Maintenance Medium containing iCell Neurons Medium Supplement (FCDI). Following an initial 100% media change at 24 h in culture, 50% media changes were then conducted every 4–7 days. The number of days in vitro refers to the days from first thawing the vial of frozen iCells and plating them on coverslips or the MEA plates.

### 2.2. Immunocytochemistry

For immunolabelling, cells were cultured on glass coverslips in 24-well plates, as described above. The cells were first washed in phosphate buffered saline (PBS) and then fixed in 4% paraformaldehyde in PBS. The fixative was aspirated, and cells were washed in PBS and incubated at 4 °C overnight in a blocking solution (5% normal donkey serum and 0.3% triton X-100 in PBS). Thereafter, cells were placed in mouse anti-β-III Tubulin primary antibody (Millipore, Burlington, MA, USA) diluted 1:500 in blocking solution and incubated at 4 °C overnight. The following day, cells were washed once in PBS and twice in blocking solution. At the completion of the final wash, cells were left in blocking solution for an additional 30 min. The blocking solution was then aspirated, and the cells incubated in a 1:200 dilution of Cy3-conjugated donkey anti-mouse IgG secondary antibody (Millipore) for 2 h at room temperature. The cells were subsequently washed in deionized water and counterstained with 100 ng/mL DAPI (Sigma-Aldrich) before mounting onto microscope slides using anti-fade mounting solution (Prolong Gold^®^ Antifade Reagent; Invitrogen, Waltham, MA, USA). Immunolabeled cells were visualized through an inverted fluorescent microscope (Nikon Eclipse TE200, Melville, NY, USA) with an attached CCD camera (PCO 1300; PCO-Tech, Willmington, DE, USA). 600–660 nm Cy3 fluorescence emission from 540–580 nm excitation was observed using a 505-nm dichroic mirror, while 435–485 nm DAPI emission from 340–380 nm excitation was observed using a 400-nm dichroic mirror.

### 2.3. Multi-Electrode Array Electrophysiology

iCell Neurons were plated at 100,000 cells per well on 24-well MEA plates (Cytoview MEA24, Axion Biosystems, Atlanta, GA, USA) previously coated with Polyethyleneimine (Sigma). Spontaneous electrical activity was acquired using the Axion Maestro Edge MEA system and Axion’s Integrated Studio (AXIS) software, v. 2.0.4.21 (Axion Biosystems) over 28 days. All recordings were performed at a constant temperature of 37 °C and 5% CO_2_. Each well of the 24-well MEA plate contains 16 individual microelectrodes, giving a total of 384 integrated recording electrodes per plate. Prior to a 10 min baseline recording period, the MEA plates were placed in the Maestro MEA platform and equilibrated for 5 min. AXIS software was used to control the heating system and to monitor the recordings, which involves simultaneous sampling of the channels at 12.5 kHz per channel with a gain of 1200× and band pass filters of 200–3 KHz. After recording, the RAW files were re-recorded with AXIS to convert the data into Microsoft Excel files (version 16), which includes spike timing and profile information. Spikes were detected using a threshold set to 6 times the estimated standard deviation of the rms-noise on each channel. Electrodes with activity higher than 0.05 spikes/s at least once over the recording time were only included for data analysis.

### 2.4. Patch-Clamp Electrophysiology

Agonist and voltage-evoked currents were recorded from cells with a neuronal-like morphology (phase bright with neurite processes), using the whole-cell configuration of the patch-clamp technique, as we have described previously (Coyne et al., 2007). Briefly, patch electrodes were made from borosilicate glass pipettes (World Precision Instruments, Sarasota, FL, USA) on a Narishige PP-830 (Amityville, NY, USA) electrode puller and had tip resistances of 2–5 MΩ. Currents were recorded using an Axopatch 200B amplifier and headstage (Axon Instruments) and low-pass filtered at 10 kHz before digitization via a National Instruments DAQ card and a National Instruments BNC-2090 interface board and stored on a computer running WinWCP software (v. 4.4.3, University of Strathclyde, Glasgow, UK). Whole cell currents were monitored on the desktop computer. Series resistance, pipette capacitance, and whole-cell capacitance were cancelled electronically.

Cells were perfused with a bath solution containing the following (in mM): NaCl (142.0), KCl (5.0), CaCl_2_ (2.0), glucose (10.0), MgCl_2_ (2.0), HEPES (5.0). The internal solution contained the following (in mM): KCl or CsCl (140.0), MgCl_2_ (2.0), EGTA (11.0), HEPES (10.0), Mg-ATP (2.0). Solutions were titrated to pH 7.4 and electrode solutions filtered before use with 0.2µm disposable filters (Millipore). All experiments were conducted at ambient room temperature (22–25 °C). Neural-like cells were voltage-clamped at a holding potential of −60 mV or current-clamped at an equilibrium potential of −70 mV, unless otherwise stated. For determination of agonist-evoked current-to-voltage relationships, the membrane potential was stepped between −120 mV and 60 mV in 20 mV increments. To activate voltage-gated currents, 20 ms voltage steps were applied from −80 to 40 mV in 10 mV increments from a holding potential of −80 mV. For recording potassium currents, CsCl was replaced with KCl or K-gluconate in the electrode solution. The bath solutions for investigating voltage-activated currents also contained 2 mM CoCl_2_, to prevent potential calcium channel activation.

### 2.5. Drugs and Their Application

All drugs were obtained from Sigma-Aldrich (St. Louis, MO, USA) except tetrodotoxin citrate (TTX), kynurenic acid, and 6,7-dinitroquinoxaline-2,3-dione disodium salt (DNQX), which were purchased from Tocris Bioscience (Ellisville, MO, USA). Stock solutions of bicuculline methyl bromide, allopregnanolone, and picrotoxin were dissolved in DMSO, while stock solutions of kynurenic acid and mefenamic acid were made up in 0.1 M sodium hydroxide. The final bath concentrations of these solvents did not exceed 0.1% of the recording solutions. Stock solutions of DNQX, phenobarbital sodium salt, chlordiazepoxide hydrochloride, d-2-amino-5-phosphonovalerate (AP5), tetraethylammonium chloride (TEA), and strychnine hydrochloride were made up in bath solution. All drug solutions were made fresh immediately prior to experiments except for TTX, which was stored at −20 °C in small aliquots of stock solution made up in deionized water. Drugs were applied directly to cells under voltage-clamp from the tip of a 250-μm pipette. Fresh bath solution was also perfused through the bath (at 1–2 mL/min) using a gravity-feed system to prevent any build-up of drug solutions in the bath. At least 2 or 3 control responses were recorded before the addition of any drug. Drugs were washed out once a stable effect was observed and control responses then re-established before further drug applications.

### 2.6. Data Analysis

Data are expressed as mean ± SEM and were analyzed by either the Student’s *t* test (two-tailed) or one-way analysis of variance (ANOVA) using Prism 9 software (GraphPad Software, San Diego, CA, USA). Tukey’s or Dunnett’s post hoc test for significance were used when variance between means was found with ANOVA. Concentration-response curves were analyzed using variable-slope nonlinear regression. Data was obtained from at least 3 individual cells per experiment, and statistical significance was set at *p* ≤ 0.05 for all analyses.

## 3. Results

**Figure 1:**
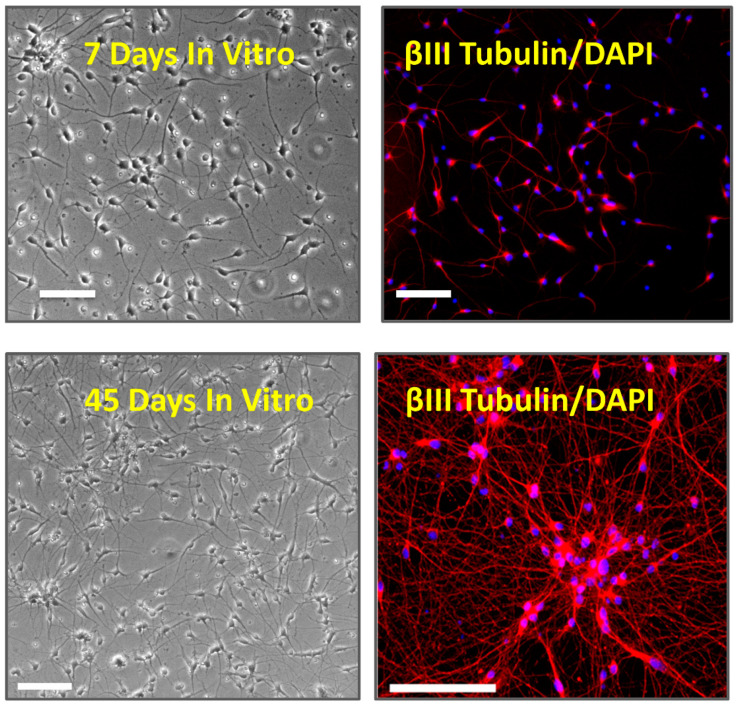
Phase-contrast image (×200) of iCell neurons (top left) and labeled for βIII Tubulin (red) + DAPI (blue, top right) at 7 and 45 days in vitro. Note the increased density of neurites and formation of cell clumping at 45 days. The phase-contrast and immunolabeled images are from different coverslips of the same iCell cultures. The calibration bars are 100 µm.

iCell neurons were successfully cultured and maintained for up to 70 days in vitro (DIV). The neurons were phase-bright with spherical or pyramidal cell bodies and multiple neurite processes that became more elaborate and denser over time in vitro. Cells stained positively with the DNA dye, DAPI, and were positive for the pan-neuronal marker βIII-tubulin, a microtubule stabilizing protein found in neuronal cell bodies and axons. We noted that cells formed small clumps or islands with longer-term culture (see Figure 1).

### 3.1. Multi Electrode Array

**Figure 2:**
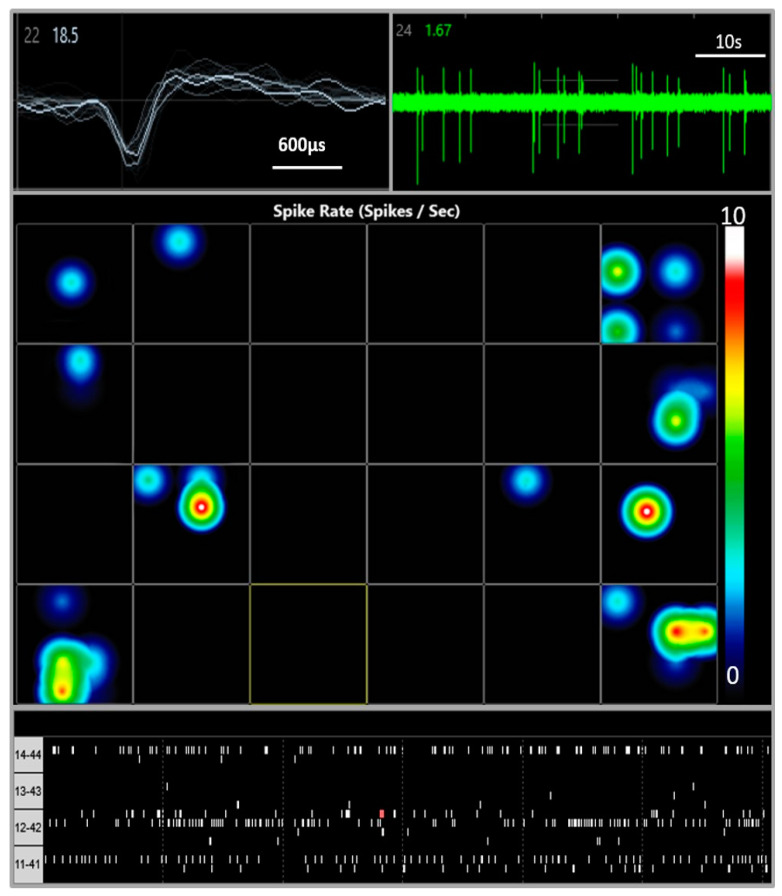
Multi-Electrode Array (MEA) recordings from iCell neurons. Top left shows extracellular voltage trace from an active electrode. Top right corner shows spike activity over baseline noise. Center panel is a color-coded heat map with a snapshot from a 24 well plate with blue indicating lower (circa 1 Hz) spike frequency and red/white high (10 Hz) activity. The bottom panel is a Raster plot showing individual electrodes and spike frequency at day 8 post-plating (with the electrode numbers shown on the left side).

**Figure 3:**
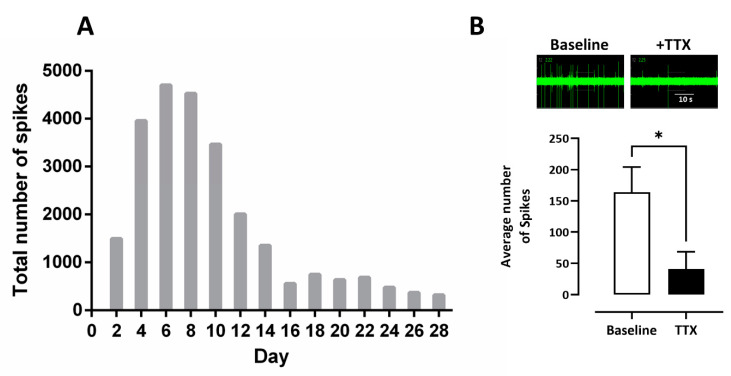
(**A**) a histogram summary of the total number of spikes recorded from iCell neurons in a 10-min recording period, every two days over 28 days in vitro. (**B**) a MEA spike trace before (baseline) and in the presence of tetrodotoxin (TTX, 0.1 µM) with a histogram showing that it significantly (* *p* ≤ 0.05) reduced the number of spikes recorded over a 10 min period. The bars and lines represent the mean ± SEM (*n* = 4).

Spontaneous electrical activity was recorded from iCell neurons cultured on MEA plates over 28 DIV. Spontaneous, spike wave pattern activity was observed within 48 h of plating across many (but not all) wells of the plate (see Figure 2) and increased over the next 8 days to a peak of 4723 spikes recorded over a 10 min sample period. This activity gradually decreased to 318 spikes recorded in 10 min at day 28 days in vitro (Figure 3 A) and did not appear to be the result of significant loss of cells on the MEA plates. In addition, we measured the spike amplitudes over 4 weeks in vitro. Maximum amplitude was observed between day 10 and day 12 with a peak of 24.4 ± 1.68 µV and then plateaued at 18.1 ± 1.06 µV by day 28 (not shown). Addition of tetrodotoxin (TTX, 0.1 µM) significantly reduced the number of spikes from 163 ± 40 (*n* = 4 wells) to 40 ± 27 (*n* = 4 wells) tested at day 28 in vitro consistent with neuronal sodium-dependent action potentials (see Figure 3B).

### 3.2. Patch-Clamp Electrophysiology

In cells sampled over the course of our experiments, the membrane potential of neurons increased from −33 ± 1.8 mV (*n* = 27) in the first week of cell culture to −41 ± 1.4 mV (*n* = 31, range −31 to −57 mV) in the fourth week of culture to −47 ± 2.2 mV (*n* = 19, range −35 to −65 mV, *p* < 0.05) in the eighth week in culture. The cell membrane capacitance recorded from sampled cells throughout ten weeks in culture was 15.1 ± 0.5 pF, the membrane resistance was 870 ± 80 MΩ and series resistance was 12.8 ± 0.5 MΩ (*n* = 95). These values are well within the ranges for immature neural cells derived from human stem cells in 2D monoculture reported by other groups [5,10,12,13].

**Figure 4:**
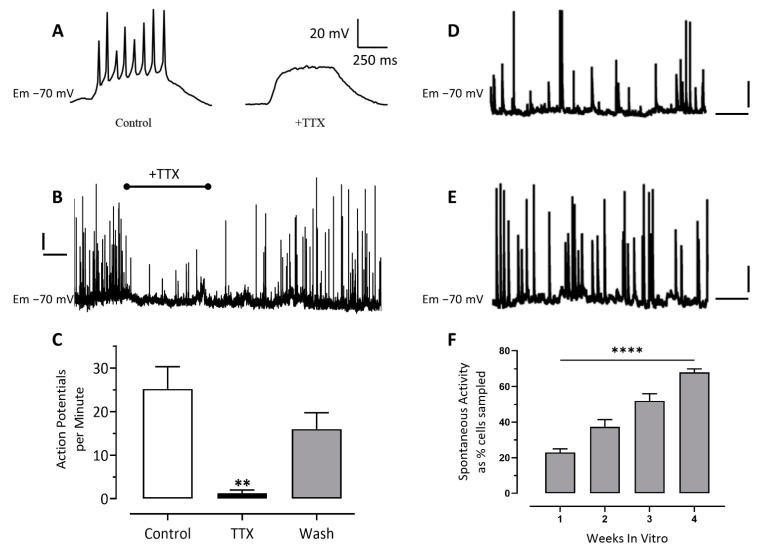
Patch-clamp recordings from iCell neurons show they fire evoked and spontaneous action potentials sensitive to tetrodotoxin (TTX). (**A**) Recording from an iCell neuron (28 days in vitro) held in current clamp at −70 mV with (0.4–5 s) depolarizing current applied to evoke a train of immature action potentials before and in the presence of TTX (100 nM). (**B**) Recording of spontaneous action potentials before, during and after washout of TTX (100 nM) to the bath solution; the calibration bars are 10 mV and 50 s. (**C**) Histogram summarizing the effect of TTX (100 nM) on the number of spontaneous action potentials per minute recorded from iCells neurons at 10 weeks in vitro. The bars and lines represent the mean ± SEM (*n* = 5). ** *p* ≤ 0.01. (**D**) Spontaneous action potentials recorded from an iCell neuron at 1 week and, (**E**) 4 weeks in vitro (the calibration bars are 20 mV and 10 s for both **D** and **E**). (**F**) Histogram showing the percentage of spontaneously active cells sampled significantly increased (**** *p* ≤ 0.0001) over 1–4 weeks in vitro. The bars and lines represent the mean ± SEM (*n* = 12–40 cells per time point).

Consistent with our MEA observations, iCell neurons held in current-clamp at −70 mV fired spontaneous action potentials (Figure 4B) that were reversibly inhibited by the sodium channel blocking drug, TTX (0.1 μM, Figure 4B,C). Action potentials could also be evoked in current-clamped cells by the injection of (0.5–0.8 s) depolarizing current (Figure 4A). These evoked action potentials were also blocked by the addition of TTX (0.1 μM, Figure 4A). Under our recording conditions the frequency of action potential discharge slightly increased (Figure 4D,E) and the proportion of cells that were spontaneously active significantly increased from 25% ± 5% in the first week of culture to 67% ± 2% in the fourth week of culture (*n* = 16 to 40 cells per week, Figure 4F).

**Figure 5:**
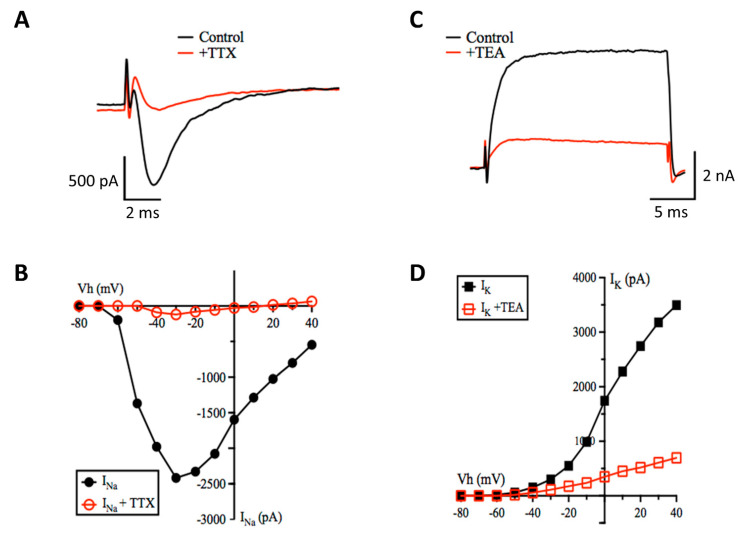
iCell neurons show robust voltage-activated currents. (**A**) whole-cell (sodium) currents evoked by a step from −80 to −40 mV (black trace) that was inhibited by TTX (100 nM, red trace). (**B**) plot of the peak inward currents against voltage steps from −80 to +40 mV in the absence (● black line) and presence (○ red line) of TTX (100 nM). (**C**) whole-cell (potassium) current evoked by a step from −80 to +40 mV (black trace) was inhibited by TEA (5 mM, red trace). (**D**) plot of the peak inward currents against voltage step in the absence (▪ black line) and presence (□ red line) of TEA (5 mM).

In a series of experiments in which the membrane holding potential was stepped from −80 to +30 mV, a fast activating and inactivating inward current and a slower activating, slowly inactivating outward current was evoked (Figure 5). Addition of TTX (0.1 μM) to the bath solution reversibly blocked the fast-inward current, consistent with the opening of sodium channels (Figure 5A), while addition of TEA (5 mM) reversibly inhibited the outward current, consistent with the activity of potassium channels (Figure 5C). The current-voltage relationships and reversal potentials of the inward and outward currents were also indicative of voltage-gated sodium and potassium channels, respectively (Figure 5B,D).

#### Inhibitory Ligand-Gated Currents

**Figure 6:**
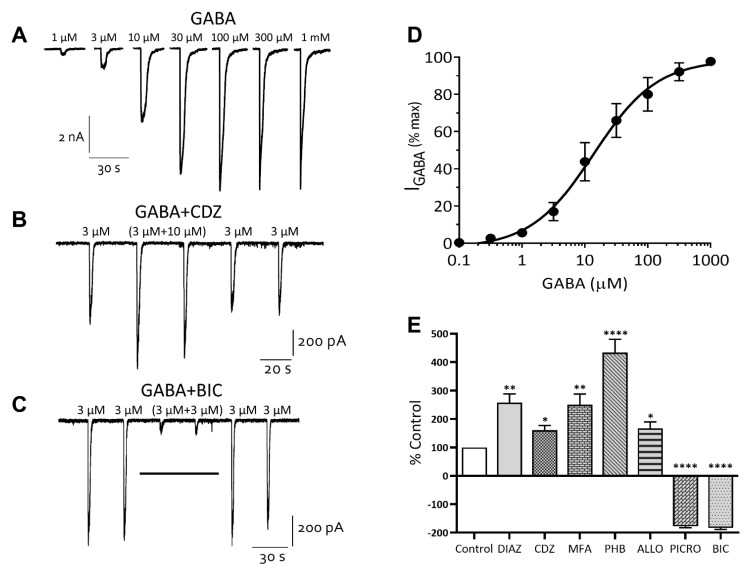
iCell neurons express GABA_A_ receptor gated chloride channels. (**A**) GABA evokes concentration-dependent currents. (**B**) sub-maximal currents are potentiated by chlordiazepoxide (CDZ) and (**C**) inhibited by bicuculline (BIC). The holding potential of cells was −60 mV. (**D**) A semi-log-linear plot of the GABA concentration-response relationship recorded from iCell neurons. The symbols represent the mean ± SEM of *n* = 11 cells. (**E**) a histogram summarizing the effects of diazepam (DIAZ, 10 µM), chlordiazepoxide (CDZ, 10 µM), mefenamic acid (MFA, 30 µM), phenobarbital (PHB, 100 µM), allopregnanolone (ALLO, 0.2 µM), picrotoxin (PICRO, 10 µM) and bicuculline (BIC, 10 µM) on sub-maximal GABA-evoked whole-cell currents. Each bar represents the mean ± SEM of 4–6 cells. * *p* ≤ 0.05, ** *p* ≤ 0.01; **** *p* ≤ 0.0001.

GABA is the most abundant inhibitory neurotransmitter in the mammalian brain, with iCell *neurons* being described as primarily GABAergic. Application of GABA to neurons under whole-cell voltage clamp evoked concentration-dependent currents with an EC_50_ of 8 μM [6–9, 95% CI] (*n* = 11) (Figure 6A,D). The GABA (3 μM) current-voltage relationship displayed a slight outward rectification and reversed at approximately 0 mV, consistent with the equilibrium potential of −1.9 mV for chloride ions under our recording conditions (not shown).

A diverse array of clinically important agents act as positive allosteric modulators of the GABA_A_ receptor and help to define it’s subunit composition [14,15]. These drugs include the benzodiazepines, barbiturates, neurosteroids and the fenamate non-steroidal anti-inflammatory drugs (NSAIDs, [4]). Addition of the benzodiazepine diazepam (10 μM) or chlordiazepoxide (10 µM, Figure 6B) potentiated GABA (EC_20_) currents to 258% ± 31% (*n* = 5) and 160% ± 15% (*n* = 5) of control, while the barbiturate phenobarbital (100 μM), the neurosteroid allopregnanolone (0.2 µM), and the fenamate NSAID, mefenamic acid (MFA, 30 μM) potentiated these currents to 433% ± 46% (*n* = 5), 180% ± 22% (*n* = 4) and 246% ± 38% (*n* = 5) of control, respectively (Figure 6E). In contrast, the non-competitive GABA_A_ chloride channel blocker, picrotoxin (10 μM) inhibited GABA (EC_60_) currents to 20% ± 5% (*n* = 4) of control and the competitive GABA_A_ receptor antagonist, bicuculline (10 µM, Figure 6C) inhibited these currents to 13% ± 2% (*n* = 4) of control (see Figure 6E).

**Figure 7:**
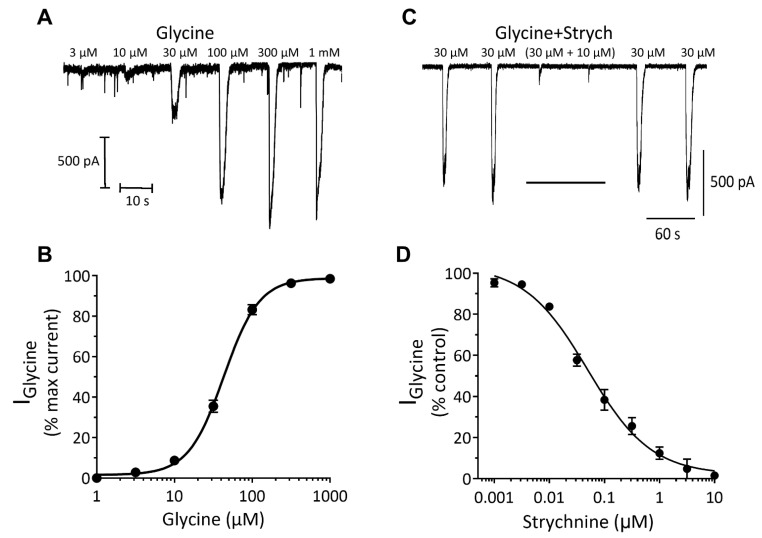
Glycine evokes robust concentration-dependent currents in iCell neurons. (**A**) glycine evoked inward currents recorded from a single iCell neuron held in voltage-clamp at −60 mV. (**B**) semi-log-linear plot of the glycine dose–response relationship recorded from iCell neurons. The symbols represent the mean ± SEM of 15 cells. (**C**) strychnine (10 μM) reversibly inhibits glycine (EC_30_) currents in a cell held at −60 mV. (**D**) a graph summarizing the effects of strychnine (0.001–10 µM) on submaximal (EC_30_) glycine evoked currents recorded from iCell neurons. The symbols represent the mean ± SEM of 4 cells.

Like GABA_A_ receptors, glycine receptors (GlyR) are anion-selective ligand-gated ion channels with activation leading to rapid increases in chloride ion permeability, membrane hyperpolarization and a reduction in neuronal excitability [16]. Here, we discovered that application of glycine evoked concentration-dependent currents with an EC_50_ of 60 μM [51–70, 95% CI] (*n* = 15) (Figure 7A,B). The glycine (30 μM) current-voltage relationship was approximately linear (Ohmic) and reversed at around 0 mV, consistent with the equilibrium potential of −1.9 mV for chloride ions under our recording conditions. Addition of the glycine receptor antagonist, strychnine led to a reversible and concentration-dependent inhibition of glycine (EC_30_) currents with an IC_50_ of 50 nM [34–74, 95% CI] (*n* = 4, Figure 7C,D). These data are consistent with those described for native glycine receptor-gated chloride currents in mammalian central nervous system neurons [16,17,18].

### 3.3. Excitatory Ligand-Gated Currents

**Figure 8:**
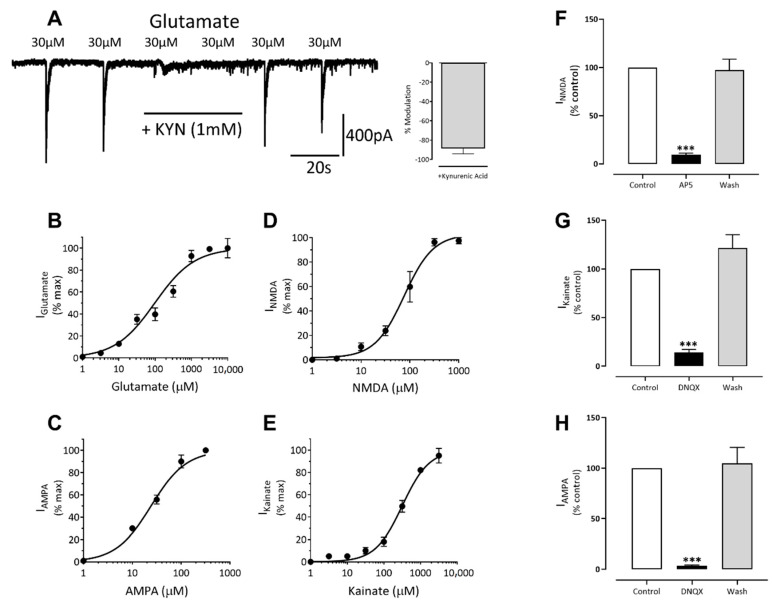
iCell neurons express ionotropic glutamate, NMDA, AMPA and kainate receptors. (**A**) A recording of submaximal (EC80) glutamate currents that are rapidly and reversibly inhibited by kynurenic acid. The histogram to the right of the trace is the mean ± SEM of 6 similar experiments. (**B**–**E**) The concentration-response curves for glutamate, AMPA, NMDA and kainate, respectively. The *x*-axis is the agonist concentration on a log scale, and the *y*-axis is the response, normalized to the maximal current. (**F**) Histogram summarizing NMDA (30 μM) responses before, in the presence, and following washout of AP5 (100 μM). (**G**) Histogram summarizing Kainate (100 μM) responses before, in the presence, and following washout of DNQX (10 μM). (**H**) Histogram summarizing AMPA (30 μM) responses before, in the presence, and following washout of DNQX (10 μM). All the bars and error lines represent the mean ± SEM of 4–6 cells; *** *p* ≤ 0.001. Neurons were voltage-clamped at −60 mV.

Glutamate is the major excitatory neurotransmitter of the mammalian central nervous system and interacts with both ligand-gated and G-protein couple receptors [19,20]. Application of glutamate (0.3–1000 µM) to neurons under whole-cell voltage clamp evoked concentration-dependent currents with an EC_50_ of 8 μM [6–10, 95% CI] (*n* = 7, Figure 8B). Addition of the broad-spectrum glutamate receptor antagonist, kynurenic acid (1 mM) reversibly inhibited glutamate (EC_80_) currents to 19% ± 6% (*n* = 6) of control (Figure 8A).

We were also able to evoke concentration-dependent currents from voltage-clamped cells using kainic acid (1–3000 µM) with an EC_50_ of 361 μM ([232–562, 95% CI] *n* = 6, Figure 8E). Additionally, α-amino-3-hydroxy-5-methyl-4-isoxazolepropionic acid (AMPA, 1–300 µM) also evoked concentration-dependent currents in voltage-clamped neurons with an EC_50_ of 21 μM [15–31, 95% CI] *n* = 4, Figure 8C). The AMPA/kainate receptor antagonist, DNQX (10 μM), reversibly inhibited kainate (EC_30_) and AMPA (EC_60_) evoked currents to 14% ± 6% (*n* = 9) and 3% ± 3% (*n* = 4) of control, respectively (see Figure 8G,H), consistent with the expression of kainate and AMPA receptors in iCell neurons.

**Figure 9:**
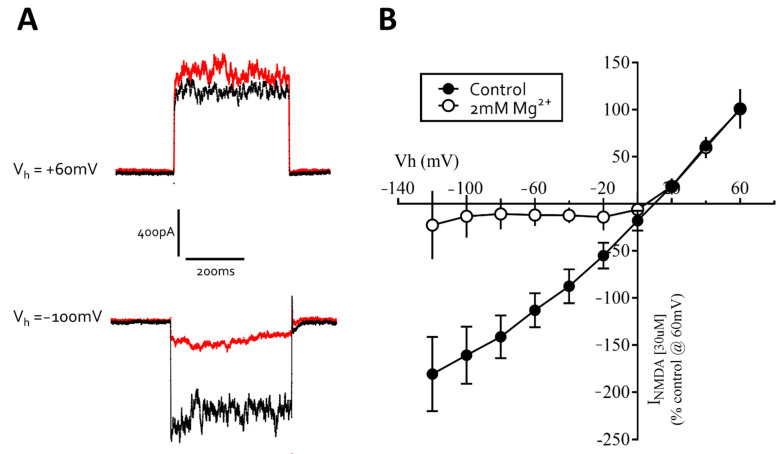
Mg^2+^ inhibits NMDA-evoked currents recorded from iCell neurons in a highly voltage-dependent fashion. (**A**) recording of responses to short (400 ms) pulses of NMDA (30 μM) applied to a neuron voltage-clamped at +60 and −100 mV in the absence (black line) and presence (red line) of Mg^2+^ (2 mM). (**B**) the current-voltage relationship for NMDA (30 μM) responses in the absence (●) and presence (○) of magnesium ions. Note the negative slope conductance above −20 mV when magnesium is included in the bath solution. The *x*-axis shows the holding potential (Vh) and the *y*-axis is the NMDA current normalized to +60 mV. The graph is the mean of 4 cells.

Application of *N*-methyl-d-aspartate (NMDA, 1–1000 µM) to neurons under whole-cell voltage clamp, with glycine (1 μM) included in the extracellular recording solution and in the absence of magnesium ions, evoked concentration-dependent currents with an EC_50_ of 57 μM [44–73, 95% CI] *n* = 4, Figure 8D). Addition of the NMDA receptor antagonist (2*R*)-amino-5-phosphonovaleric acid (AP5, 100 μM) reversibly inhibited NMDA (EC_30_) currents to 8% ± 3% (*n* = 4) of control (Figure 8F). In the absence glycine, NMDA currents were diminished below *c.* 25 pA confirming that glycine is a required co-agonist. We also determined that the NMDA (30 μM) current-voltage relationship was approximately linear in the absence of Mg^2+^ but displayed strong voltage-dependent block at negative potentials, with a region of negative slope conductance above −20 mV holding potential (Figure 9A,B). Together, our data show that iCell Neurons express functional NMDA-type glutamate receptors with several of the complex pharmacological properties described for native CNS neuron receptors.

## 4. Discussion

Human iPSC-derived neurons are increasingly promoted as tools in drug discovery (e.g., [6,21,22,23], even when their molecular pharmacological properties are not fully characterized [5]. We have previously reported on the electrophysiological characteristics of neurons derived from a human stem cell line that is simple and inexpensive to culture and differentiate [24,25]. Here, we determined the physiological and pharmacological properties of the voltage- and ligand-gated ion channels expressed in a commercially available population of iPSC-derived neurons (iCell neurons) maintained for up to 10 weeks in vitro, using both multi-electrode array and patch-clamp approaches.

Our data showed that iCell neurons display neurite outgrowth within 24 h of plating and label for the pan-neuronal marker, βIII tubulin within the first week consistent with the morphological and immunocytochemical features of neurons. Our MEA recordings also showed that these iPSC-derived neurons generated spontaneous spike-like activity within 2 days of plating which peaked after one week, and then rapidly decreased over the second week to remain at low levels up to one month in vitro. The reduced spike activity was not associated with a coincidental rapid loss of cells from the MEA plate. A previous study that used MEA to record from iCell neurons for 9 days post-plating also reported they were most active at day 7 to 8 [26] consistent with our recording from these cells over 28 days post-plating. Odawara and colleagues (2014) similarly reported that spontaneous activity rapidly decreased in iCells after 8 days in vitro unless co-cultured with rat astrocytes or in glial cell conditioned media [27]. The extracellularly recorded spikes were inhibited by the sodium channel blocker, tetrodotoxin, consistent with the generation of neuronal action potentials, and in keeping with a short report by Kasteel and colleagues (2017) [28]. Together, these data indicate that this population of iPSC-derived neurons, in standard culture conditions, may be useful for shorter-term experiments, particularly when spontaneous neuronal activity is a focus of research.

Our patch-clamp recordings showed that the passive membrane properties of iCells neurons, including their membrane capacitance, membrane resistance and membrane potential are consistent with native neurons [29], embryonic stem cell derived neurons (e.g., [12]), and iPSC-derived neurons (e.g., [10,30]). Cells also generated evoked and spontaneous action potentials within the first week of plating, which increased over one month in culture, along with a significant increase in the proportion of cells that were spontaneously active, indicating that such pre-differentiated neurons continue to mature in vitro. Our single cell recordings showing increased spiking over the first month therefore differed from our MEA recordings showing a decrease. The decrease in spontaneous activity, around 6–8 days in vitro is likely the result of some migration of cells on the MEA chip (see also Odawara et al., 2014) but, most significantly, the development of a dominant inhibitory tone from dense GABAergic neuron circuit maturation suppressing spontaneous spike activity. In support of this hypothesis, FCDI reported that the GABA_A_ receptor antagonist, GABAZINE increased bursting rate in iCell neurons (https://fujifilmcdi.com/assets/CDI_iCellNC_AxionMEA_AP-NRCAXN1405281.pdf accessed on 28 July 2021). In supplementary experiments (Supplementary Figure S1) we determined that iCell neurons, under current clamp, and depolarized by glutamate, leads to the generation of only a few action potentials, even in the continued presence of glutamate. In contrast, pre-applying the GABA_A_ receptor antagonist, bicuculline (to block inhibitory [GABA] tone) and then evoking depolarization with glutamate leads to a train of action potentials that are well maintained throughout application of glutamate (see Supplementary Figure S1) These data therefore strongly support the hypothesis that iCells are under powerful GABAergic inhibitory tone and the reason for the reduction in spike activity beginning around 6–8 days on the MEA plate. The voltage-step protocols also confirmed that both voltage-gated sodium and potassium ion channels are strongly expressed, can be blocked by TTX and TEA, respectively, and underlie the recorded action potentials, consistent with previous reports [9,10,28]. Our observations support the value of iCell neurons as a model for studies of neuronal voltage-gated sodium and potassium channels, and the study of glutamate-evoked action potentials.

In addition, our patch-clamp recordings revealed several important neuropharmacological properties of iCells. First, neurons responded to the application of GABA (in keeping with the manufacturers (re)designation of iCells as iCell GABANeurons) from 2 days post-plating and for a further 3 months in vitro. Previous studies using automated patch-clamp instruments to record from ensembles of cells reported that GABA currents in iCells were inhibited by bicuculline [9] and modulated by diazepam [11]. In the present study, we show that GABA responses were reversibly inhibited by both the competitive and the non-competitive GABA_A_ antagonists, bicuculline and picrotoxin, respectively. Moreover, we demonstrate for the first time that these GABA currents are potentiated by a diverse range of positive allosteric modulators of the GABA_A_ receptor including chlordiazepoxide, diazepam, phenobarbital, allopregnanolone and mefenamic acid. Inhibition of GABA currents by bicuculline and picrotoxin indicates a receptor conformation to include β-α subunits forming the GABA and bicuculline binding site as well as the transmembrane domain site for picrotoxin [31]. Sensitivity to the benzodiazepines, chlordiazepoxide and diazepam, is conferred by the presence of the γ2 receptor subunit in the GABA_A_ receptor complex [32,33] whilst sensitivity to the NSAID, mefenamic acid is conferred by the presence of the β2/β3 subunits in the receptor complex [4,34]. We can therefore deduce from our electrophysiological data that the GABA_A_ receptor isoforms expressed by iCells are likely composed of αxβ2/3γ2 subunits (where x is one of several possible α subunits) to enable the rich and complex pharmacological responses observed to these clinically important agents and consistent with native GABA_A_ receptor pharmacology [35]. Measurements of mRNA expression levels of GABA_A_ receptor subunits in iCell neurons are also entirely consistent with our patch-clamp data [7,11]. Together these data indicate that these iPSC-derived human neurons may be valuable models for studies of the GABA_A_ receptor chloride channel complex in drug discovery and development.

This study also revealed for the first time that the inhibitory neurotransmitter glycine evoked robust and concentration-dependent responses in iCell neurons and that these currents were inhibited by the competitive antagonist of glycine receptors, strychnine [36]. Like GABA_A_ receptors, glycine receptors are members of the Cys-loop receptor family and mediate synaptic inhibition in the spinal cord, brainstem, and other caudal regions of the brain [37]. Glycine receptors are involved in nociception and implicated in several neurological diseases, including hyperekplexia (startle disease) and autism; they are inhibited by drugs such as picrotoxin and potentiated by general anesthetics, ethanol and endogenous cannabinoids [38]. iCell neurons may therefore serve as a useful tool to investigate the pharmacological properties of human glycine receptors.

Finally, we determined extended agonist concentration range responses to glutamate, AMPA, kainate and NMDA from iCell neurons, providing new information on saturating and non-saturating agonist concentrations that are essential for studies of positive and negative allosteric modulators of these major excitatory receptors (e.g., [8]). Our data are consistent with the manufacturer’s description that iCell neurons are a mixed population of post-mitotic neural subtypes comprised of both GABAergic and glutamatergic neurons. The agonist-evoked currents were inhibited by antagonists consistent with the expression of the ionotropic AMPA, kainate and NMDA glutamate receptors. Additionally, we determined that the NMDA currents, like native neuronal receptors, were inhibited in a highly voltage-dependent manner by magnesium ions [39] and required the presence of the co-agonist, glycine in the extracellular recording solution [40]. Consistent with our current findings, Dage and colleagues (2014) showed that application of AMPA or NMDA evoked transient calcium fluxes in iCell neurons. In addition, and based on their gene expression profile, the NMDA receptors expressed in iCell neurons were reported to be composed of GluN1 and GluN2B heterodimeric subunits, similar to that found in neonatal cortex [7,8]. Overall, these data are therefore consistent with the many of the physiological and pharmacological properties of those described for native ionotropic glutamate receptors [20,41].

In summary, our electrophysiological observations on iCell neurons show they express several of the major voltage- and ligand-gated ion channels with the neurophysiological and neuropharmacological properties consistent with native neurons. We also show that these cells remain viable in long-term culture for experimentation. These new data therefore suggest their value for pharmacological research and drug development, especially given the role of GABA_A_ and ionotropic glutamate receptors in normal brain functions of fast synaptic neurotransmission and synaptic plasticity to pathological conditions such as epilepsy, anxiety, depression, neurodegeneration and stroke [41,42]. Moreover, recent studies have indicated that iPSC-derived neurons and glial cells may also have value and greater sensitivity than rodent neurons in neurotoxicology studies [43,44,45]. Future work in this lab, however, will determine the functional properties of iPSC-derived neurons and glia in 3D cerebral neurospheres (brain organoids) in an effort to better understand their validity as more complex models of the human nervous system in vitro.

## Data Availability

Data available on request.

## Supplementary Materials

The following are available online at https://www.mdpi.com/article/10.3390/cells10081953/s1, Figure S1: (A) A single iCell neuron held in current clamp at −70 mV, transiently exposed to glutamate (30 μM) depolarizes the cell and evokes 4–5 spikes. (B) A single iCell neuron with bicuculline (3 μM) included in the bath solution transiently exposed to glutamate (30 μM) evokes depolarization and a train of action potential throughout the membrane depolarization. (C) A histogram of the number of action potentials recorded over 100 s when cells are depolarized in the absence (control) or presence of bicuculline.
