# Supplementary material for: An Electrophysiological and Pharmacological Study of the Properties of Human iPSC-Derived Neurons for Drug Discovery
Source: Cells. 2021 Jul 31;10(8):1953. doi: 10.3390/cells10081953 (PMC8395001; doi:10.3390/cells10081953)
Supplement: Supplementary file 1 [file cells-10-01953-s001.zip › cells-1295875-supplementary.pdf]

cells

MDPI

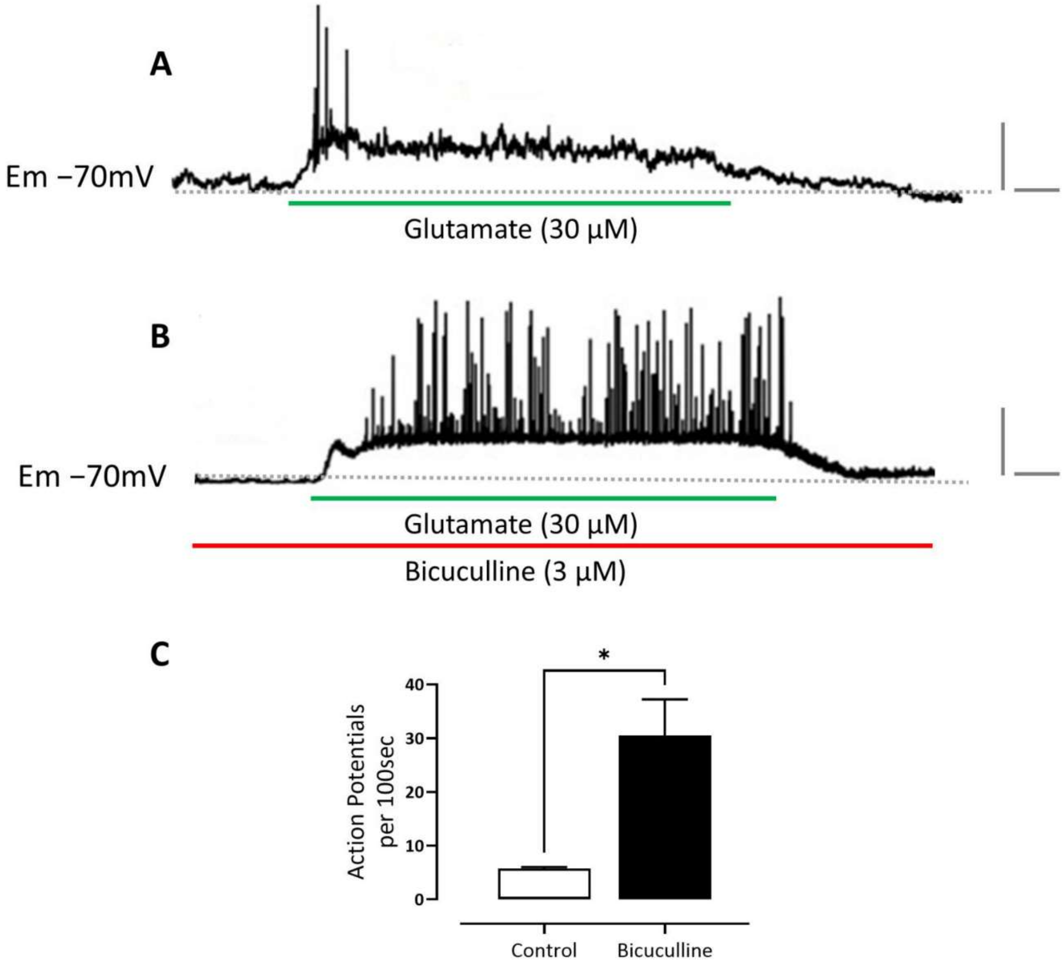

Figure S1. (**A**) A single *iCell* neuron held in current clamp at −70 mV, transiently exposed to glutamate (30 μM) depolarizes the cell and evokes 4–5 spikes. (**B**) A single *iCell* neuron with bicuculline (3 μM) included in the bath solution transiently exposed to glutamate (30 μM) evokes depolarization and a train of action potential throughout the membrane depolarization. (**C**) A histogram of the number of action potentials recorded over 100 s when cells are depolarized in the absence (control) or presence of bicuculline. As can be seen, bicuculline significantly (* $p \leq 0.05$) increased the number of action potentials. The bars and lines represent the mean ± sem of $n = 4$. The bath solution was Mg^{2+}-free; the internal solution was potassium gluconate-based. The time and voltage calibration bars = 25 s and 50 mV respectively. *iCells* were 9–10 weeks in vitro.
